# Supplementary material for: Improved Pharmacokinetics of Icariin (ICA) within Formulation of PEG-PLLA/PDLA-PNIPAM Polymeric Micelles
Source: Pharmaceutics. 2019 Jan 25;11(2):51. doi: 10.3390/pharmaceutics11020051 (PMC6409701; doi:10.3390/pharmaceutics11020051)
Supplement: Supplementary file 1 [file pharmaceutics-11-00051-s001.pdf]

pharmaceutics

Supplementary Materials: Improved Pharmacokinetics of Icariin (ICA) within Formulation of PEG-PLLA/PDLA-PNIPAM Polymeric Micelles

Lu-Ying Han, Yun-Long Wu, Chun-Yan Zhu, Cai-Sheng Wu and Chun-Rong Yang

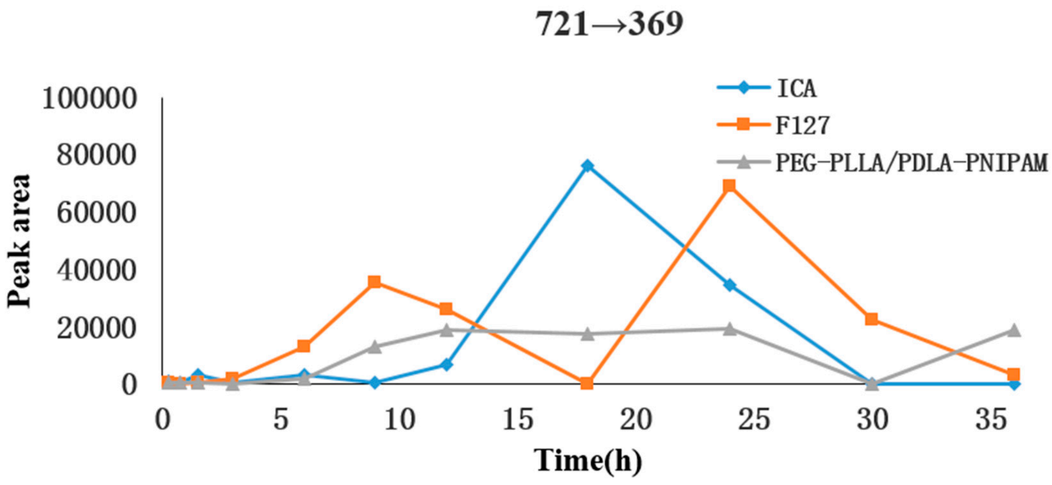

(A)

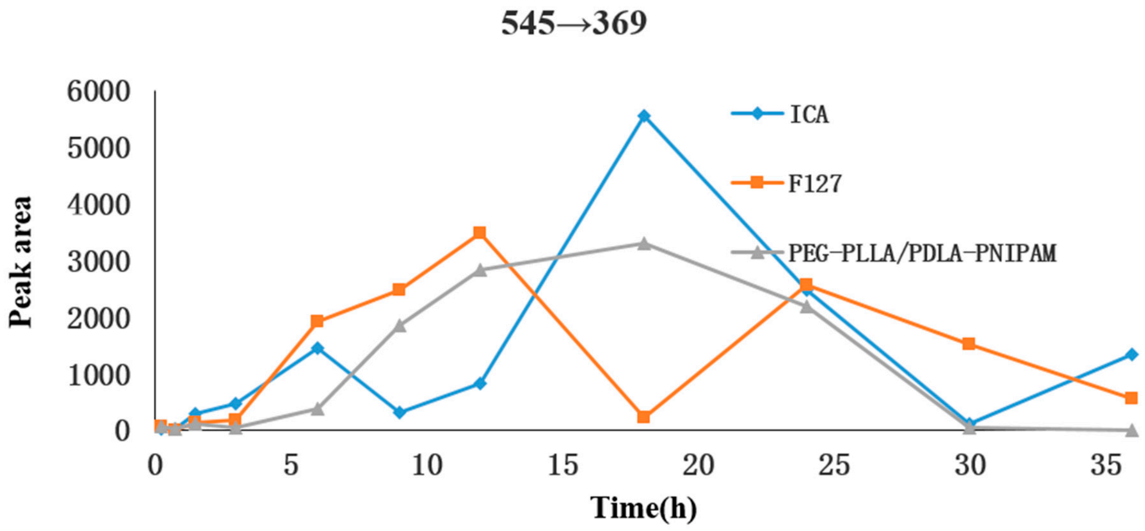

(B)

Figure S1. ICA in vivo metabolite product line chart. (**A**) 721→369; (**B**) 545→369.
